# Spatial Distribution and Risk Assessment of Heavy Metal(oid)s Contamination in Topsoil around a Lead and Zinc Smelter in Henan Province, Central China

**DOI:** 10.3390/toxics11050427

**Published:** 2023-05-03

**Authors:** Ling Yang, Shiji Ge, Jinhui Liu, Younas Iqbal, Yuling Jiang, Ruiling Sun, Xinling Ruan, Yangyang Wang

**Affiliations:** 1 National Demonstration Center for Environmental and Planning, College of Geography and Environmental Science, Henan University, Kaifeng 475004, China; yangling2009@henu.edu.cn (L.Y.); GSGe@henu.edu.cn (S.G.); ljh123@henu.edu.cn (J.L.); younasiqbal48@yahoo.com (Y.I.); 2 Key Laboratory of Geospatial Technology for the Middle and Lower Yellow River Regions, Henan University, Ministry of Education, Kaifeng 475004, China; 3 Henan Engineering Research Center for Control & Remediation of Soil Heavy Metal Pollution, Henan University, Kaifeng 475004, China; 4 School of Geographic Sciences, Xinyang Normal University, Xinyang 464000, China; kf_jyl2019@163.com; 5 Puyang Branch of Municipal Bureau of Ecological Environment, Puyang 457100, China; sunrl780503@163.com

**Keywords:** heavy metal(oid)s, lead/zinc smelter, spatial distribution, risk assessment

## Abstract

A total of 137 farmland soil samples were collected around a lead/zinc smelter within 64 km^2^. The concentration, spatial distribution, and potential source of nine heavy metal(oid)s (As, Cd, Co, Cr, Cu, Ni, Pb, V, and Zn) in soils and their potential ecological risk were investigated in detail. The results showed that the average concentrations of Cd, Pb, Cr and Zn in these soils were higher than their background value in Henan Province, and the average content of Cd was 2.83 times of the risk screening values in the national standard of China (GB 15618-2018). According to the distribution of different heavy metal(oid)s in soils, Cd and Pb in soil decrease gradually with the increase of distance from the smelter to the surrounding area. This indicates that the Pb and Cd originate from smelters via airborne practices according to the typical air pollution diffusion model. The distribution of Zn, Cu, and As were similar to Cd and Pb. However, Ni, V, Cr, and Co were mainly affected by soil parent materials. The potential ecological risk of Cd was higher than those of other elements, and the risk grade of the other eight elements was mainly low. The polluted soils with significantly high and high potential ecological risk covered 93.84% of all the studied regions. This should be of serious concern to government. The results of a principal component analysis (PCA) and cluster analysis (CA) show that Pb, Cd, Zn, Cu, and As were the elements mainly stemmed from smelter and other types of plants, with a contribution rate of 60.08%, while Co, Cr, Ni, and V are mainly caused by nature, with a contribution rate of 26.26%.

## 1. Introduction

Soil is a fundamental resource for humanity, yet anthropogenic activities have resulted in substantial soil contamination [1,2,3]. Pollutants such as heavy metal(oid)s [3,4,5], oil [6], pesticides [7], and antibiotics [8] have been identified as significant contributors to this issue. Among these, heavy metal(oid)s are particularly ubiquitous, toxic, persistent, resistant to biodegradation, and capable of bioaccumulation in the food chain [9], posing a significant threat to human health and the ecosystem [3]. The assessment of heavy metal pollution status in soil has been a major focus of researchers, with investigations conducted on urban soils [10], roadside soils [11], soils in urban parks [12], and urban road dusts [13]. Heavy metal(oid)s can be released into the environment through natural and anthropogenic sources, with industrial production processes being a significant contributor [14,15,16]. Consequently, some researchers have concentrated on developing remedial methods for heavily polluted soils containing heavy metal(oid)s [17,18,19,20,21].

Some scholars have investigated the impact of smelting on the proliferation of heavy metal(oid)s in the surrounding soil [15,22,23] due to metal processing and manufacturing. The metals released during smelting can migrate to various environmental media including airborne particles [23], dust [24,25,26], gas [27,28], sewage [29], and solid waste [30], with soil being the most heavily impacted [31]. Wu et al. [32] reported that 99.1% of agricultural soil samples around a Pb/Zn mining and smelting area in southwest China were highly polluted or worse by Pb, Zn, and Cd. Atmospheric deposition is believed to be a significant contributor to the transport of heavy metal(oid)s to soil farther away [23,24,25,26,27,28,33]. The monsoon and wind directions influence the migration and sedimentation of the smelter waste gas to soil around the smelters [28].The high pollution from smelters is a great health risk to humans [33,34,35]. However, little research has been conducted on the effect of smelting on heavy metal(oid)s contamination in soil in Henan Province (China).

Henan Province, situated in central China, is a significant contributor to the production of wheat grain, with the highest yield in the country and accounting for approximately 25% of the national output. The quality of wheat grain produced in Henan Province has a substantial impact on China’s food security. Cheng et al. [36] studied the cultivated soils of Henan Province, with moderate and moderate-to-strong contamination from Mn and Zn. In a soil sample from a small watershed in the mountainous area of southern Henan, China, 68.55% of the topsoil in the study area was clean, with Hg found to be the main contamination element [37]. Previous research has demonstrated a positive correlation between the total Cd content in soil and the concentration of Cd in wheat grain near mining areas and plants [38,39,40]. Therefore, it is vital to conduct investigations into the impact of smelters on heavy metal(oid)s in agricultural soils in Henan Province.

The aims and objectives of the current investigation were threefold: (a) to examine the extent of contamination and spatial distributions of As, Cd, Co, Cr, Cu, Ni, Pb, V, and Zn in agricultural soil surrounding the Pb/Zn smelter; (b) to evaluate the potential risks associated with heavy metal(oid)s pollution; and (c) to analyze the contribution of various sources of heavy metal(oid)s’ pollution to soil in the vicinity of the smelter.

## 2. Materials and Methods

### 2.1. Study Area

**Figure 1:**
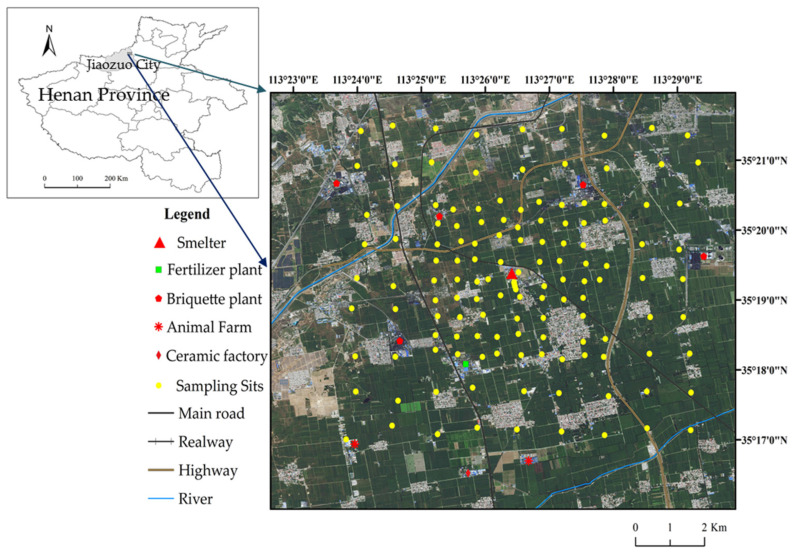
Location of the study area and the sampling sites.

The research site, situated in the northeast of Jiaozuo City in Henan Province, central China, approximately 22.4 km away, centered around the Dongfang Pb/Zn smelter (33°19′17.61″ N, 113°02′17″ E) is now closed (Figure 1). The Dongfang Gold Lead Co., Ltd. in Jiaozuo, China established the smelter in 2003 and produced lead, zinc oxide, gold, silver, copper, and indium. The smelter had a yearly production capability of 60,000 metric tons of electrolytic lead, 35,000 metric tons of sulfuric acid, 150 metric tons of silver, 0.5 metric tons of gold, and other commodities. Nevertheless, due to inadequate ecological management, the smelter discharged an extensive amount of pollutants into its surroundings during production. As a result, the local government closed the smelter in 2014. The study site encompasses villages, farmlands, highways, railways, and factories. It has a typical continental monsoon climate characterized by four distinct seasons, with an annual average temperature of 14.8 °C and a precipitation of 652 mm. The soil in the study area was classified as Luvisol and Fluvisol [41]. The primary crops grown in this area include wheat, corn, and vegetables, which serve as important food production bases for local residents and nearby markets.

### 2.2. Sampling Procedure

Soil samples were collected from a 64 km^2^ area around the Dongfang Pb/Zn smelter. Sampling sites were laid at intervals of 0.5 km around the smelter within 2 km and at intervals of 1 km within the range of 2–4 km around the smelter. Most of these samples were taken from agricultural fields, with just a few soil samples collected from grasslands and forests. The main crops in this area are wheat and maize. The sampling sites are shown in Figure 1. A total of 137 topsoil samples (0–20 cm depth) were collected around the smelter. To minimize heterogeneity and uncertainty, soil was sampled from five locations at each site within 5 m, and the collected samples were put into polyethylene bags, labeled, and transported to the laboratory. Samples were thoroughly mixed together, air-dried and manually crushed to 18 mesh size (1 mm) and 100 mesh size (0.15 mm) for detection of soil chemical properties and heavy metal contents.

### 2.3. Chemical Analysis

Soil pH was determined using a pH meter equipped with a glass electrode, at a soil to water ratio of 1:2.5 (*w*/*v*). Soil organic matter (OM) was analyzed by employing the wet oxidation technique with K_2_Cr_2_O_7_ and concentrated H_2_SO_4_, as outlined by Bi et al. [42]. Total nitrogen was quantified by the Kjeldahl method following digestion with H_2_SO_4_. Cation exchange capacity (CEC) was assessed using the ammonium acetate method. Total metal concentrations (Cd, Co, Cr, Cu, Ni, Pb, V, and Zn) were measured using ICP-AES (Thermo Fisher Dou6200, Waltham, NA, USA) after digestion with mixed strong acid HNO_3_, HF and HClO_4_ [43]. The As concentrations were determined using AFS (Haitian 3100, Haiguang Corp., Beijing, China), following the protocol outlined in our earlier publication [44]. Quality assurance and quality control were ensured by employing the standard reference material of ESS-2 from China, with the recovery of the nine heavy metal(oid)s ranging between 95% and 110%. The practical limits for the analysis of Cd, Co, Cr, Cu, Ni, Pb, V, and Zn were 0.003, 0.005, 0.004, 0.005, 0.008, 0.03, 0.004, 0.006 mg/L, respectively. Duplicated samples for each metal were analyzed simultaneously, with standard deviations within 5%.

### 2.4. Potential Ecological Risk (PER) Assessment

Potential ecological risk (PER) has been widely used for soil and sediment pollution assessment [45]. The PER index is calculated as follows [46]:

(1) $$PER=\sum_{i=1}^{n}E_{r}^{i}=\sum_{i=1}^{n}\left(T_{r}^{i}\times C_{r}^{i}\right)=\sum_{i=1}^{n}\left(T_{r}^{i}\times \frac{C^{i}}{C_{n}^{i}}\right)$$

where *PER* is the potential ecological risk caused by the overall contamination, $E_{r}^{i}$ is the potential ecological risk index caused by individual heavy metal, $T_{r}^{i}$ is the biological toxic factor of an individual element, which is defined as: Zn = 1, V = Cr = 2, Cu = Co = Pb = 5, Ni = 6, As = 10, Cd = 30 [32,33,34]. $C_{r}^{i}$ is the individual element pollution factor; the value is $C^{i}$ divided by $C_{n}^{i}$, with $C^{i}$ being the concentration of element in soil samples, and $C_{n}^{i}$ the reference value of the element. The background concentration of each metal in the Henan Province was used as the reference value. The division and class of *E_r_* and *PER* were defined in Table 1 [47].

### 2.5. Statistical Analysis

The descriptive statistics, origin data analysis, and PER calculation were executed using Microsoft Excel software 2010 (Microsoft, Redmond, WA, USA). Multivariate statistical techniques, including a principal components analysis (PCA) and cluster analysis (CA), were examined using SPSS 25.0 (IBM, New York, NY, USA). The spatial distribution of soil heavy metal(oid)s and PER was produced utilizing the ArcGIS 10.07 (ESRI, Redlands, CA, USA) software, employing the spatial analyst mode.

## 3. Results and Discussion

### 3.1. Heavy Metal Contamination

The chemical properties, such as pH, OM, total nitrogen (TN), CEC, and Fe, concentration of these soil samples around smelters are shown in Table 2. The average pH of the soil samples was 8.55, indicating that the soil in this area is alkaline. The average OM, CEC, and TN were 2.57%, 22.91 cmol/kg, 0.21%, respectively, which means the soil is still relatively fertile.

The mean concentrations of As, Cd, Co, Cr, Cu, Ni, Pb, V, and Zn were 17.34, 2.6, 15.97, 101.07, 18.28, 9.31, 67.26, 45.95, and 72.18 mg/kg, respectively (Table 3). The mean concentrations of Pb, Cd, As, Zn, and Cr in these soil samples were all higher than that of the background value of Henan Province. However, the mean concentrations of Cu, Ni, and V were not higher than the background value. Among these metals, the mean concentration of Cd was four times higher than the risk screening values for soil contamination of agricultural land in China (0.6 mg/kg, GB15618-2018). Further, the Pb, Cd, and As content in some soil samples also exceeded the risk screening values of China, and the excess rate reached 8.03%, 94.89%, and 10.22%, respectively (Table 3). This shows that Cd is the most polluting element in soils around the smelter. This result implies that the Pb/Zn smelter has had a great impact on the content of heavy metal(oid)s in the surrounding soil, and the heavy metal(oid)s formed during the production process were distributed via sewage, aerosols, and dust [26]. The mean content of Pb in soil in the present study was twice as high than that in a study reported by Li et al. in Hunan province [48], although the As and Cd content were similar. 

Table 3 presents the coefficients of variation (CV) for Pb and Cd, which were found to be 167.77% and 165.18%, respectively. These values indicate strong variations, defined by a CV greater than 100%. These significant changes in soil composition are likely the result of smelter discharge and other anthropogenic sources. Conversely, Zn, Cu, and Ni exhibited CVs between 40% and 70%, indicating moderate variations and suggesting little artificial influence by these elements. Furthermore, CVs for Cr, Co, V, and As were less than 40%, indicating weak variations. The findings suggest that these elements are influenced primarily by more uniform local parent materials without anthropogenic influence.

### 3.2. Spatial Distribution of Heavy Metal(oid)s

**Figure 2:**
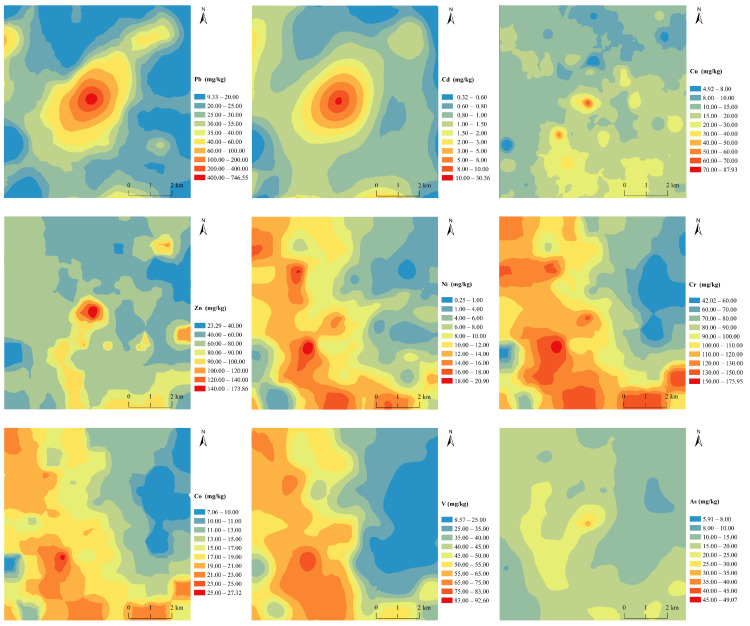
The spatial distribution of heavy metal(oid)s in topsoil near the Pb/Zn smelter.

Precise spatial distribution maps of heavy metal(oid)s are an essential key to reducing the negative impacts on the ecosystem and residents [50]. According to Figure 2, the spatial distribution of heavy metal(oid)s can be classified into three categories based on the influence of the smelter. First, the concentration of Pb and Cd gradually decreases as distance from the center of the smelter increases. However, in the study area, only the west, east, and north areas meet the national soil environmental quality standard of 0.6 mg/kg, indicating that local soil has been severely contaminated by Cd. Furthermore, within a 0.5 km radius of the smelter, the Pb content exceeds the risk screening values of 170 mg/kg. Field surveys reveal that the soil in the study area was not irrigated with sewage and that there was no haphazard piling of smelting slag. Thus, it can be inferred that the presence of Pb and Cd in the soil was due to the emission of smoke and dust from the smelter, which settled near the smelter through atmospheric sedimentation [24]. The distribution of Pb and Cd takes on an oval shape that extends from northeast to southwest, which is likely linked to the southwesterly wind direction. Zhou et al. [22] reported that heavy metal(loid)s were mainly deposited within a 2 km distance to the smelters. In this study, the area influenced by Cd is larger than 4 km, Pb is 3 km, while the other elements are less than 2 km. In the process of atmospheric migration, with the increase of distance, the difference of the mean concentrations of the easily diffusible heavy metals becomes smaller [27].

Second, the concentrations of Zn, Cu, and As in the soil gradually decreased with increasing distance from the smelter, with a less pronounced decrease trend observed for Zn, Cu, and As compared to Pb and Cd. This indicate that Cd diffused more easily into a larger area than Zn, and farther away from the smelter [27]. The concentration of As in the soil near the smelter exceeded the national standard value of 25 mg/kg, but gradually decreased as the distance from the smelter increased. Regions with higher Cu concentrations were mainly concentrated in the southeast of the study area, and these regions were found to be consistent with the locations of chemical fertilizer plants and animal farms (Figure 2). It is inferred that the pollution sources of Cu in the study area are the chemical fertilizer plants and animal farms, which contribute to the overall increase of Cu in the soil. Previous research has reported that the average Cu concentration in pig feed sold in China has reached 200–300 mg/kg [51]. Additionally, aquaculture wastewater from the farms is commonly used for sewage irrigation, and animal manure is used as organic fertilizer in farmland, which is the primary reason for the high concentration of Cu in the soil surrounding the animal farms.

Third, the spatial distribution of Ni, V, Cr, and Co within the study area exhibited noticeable similarity, characterized by a distinct zonal pattern with elevated concentrations in the northwest-southeast along with the presence of 2–3 high-value centers. This distribution pattern is primarily attributable to the presence of the provincial highway (S233) traversing the area from north to south, as shown in Figure 1. Notably, the highway is surrounded by several industrial facilities such as fertilizer and briquette plants, among others. Soils in close proximity to the highway exhibited slightly higher concentrations of Ni, V, Cr, and Co, with the highest values recorded in the vicinity of the chemical fertilizer plant located in the southwest. The concentrations of these elements in the study area were relatively low, falling below national standards, with the lowest concentrations recorded in the northeastern region of the study area.

In general, the smelter has a significant impact on the concentration of Cd and Pb in the soil, particularly Cd. The distribution of these metals in the vicinity of the smelter displays a clear pattern of point source pollution diffusion model, with concentrations decreasing gradually with distance from the smelter. This phenomenon may be attributed to the deposition of atmospheric particulate matter from the smelter, which then settles in the surrounding area. The dispersion of Zn, Cu, and As is also influenced by the smelter, albeit to a lesser extent, with a limited range of influence. Conversely, the presence of Ni, V, Cr, and Co in the soil is largely independent of the smelter, and is instead influenced by other local industries, such as fertilizer and animal plants.

### 3.3. Assessment of Potential Ecological Risk (PER)

The potential ecological risk index of heavy metal(oid)s in soils is shown in Table 3. Notably, the E_r_ for both Pb and Cd in all soil samples exhibit substantial variance, with Cd displaying the largest range. Relative to the other heavy metal(oid)s, Cd has the most significant range and mean E_r_, followed by Pb, As, Co, Cu, Cr, V, and Zn. The mean E_r_ of Cd exceeds the mean value of the other heavy metal(oid)s by 500–10,000 times, a trend attributed to the higher toxicity response coefficient of Cd. Further, this finding underscores that the Cd levels in soil samples around the smelter are generally higher than the background value. The PER of the nine toxic elements in all the soil samples ranges from 146.86 to 12,549.81, with an average of 1107.18, indicating a high overall ecological risk in the study area (Table 4).

The percentage of potential ecological risk in different grades for each of the heavy metal(oid)s is shown in Table 5. For Cd in all sampling sites, 0.73%, 15.33%, and 83.94% of the sites had considerable risk, high risk, and very high potential ecological risk, respectively. For Pb, 91.97% had low risk, 2.19% had moderate risk, 5.11% had considerable risk, and 0.73% of the sites had high risk. For As, 1.46% had moderate risk. Conversely, the potential ecological risks of Zn, Cu, Ni, Cr, Co, and V in all soil samples were low risk (Table 5). These results indicate that Cd in soil posed the greatest risk to the ecosystem in the study area.

Based on the PER values pertaining to heavy metal(oid)s, it was observed that 40.15%, 51.09%, and 8.03% of the soil samples were associated with high, considerable, and moderate risks, respectively. The total of these three risk levels amounted to 99.27%, thereby suggesting that nearly all the soil within the study area has been exposed to some degree of hazard. Additionally, the contribution rate of Cd to PER in the soil samples ranged from 84.51% to 98.57%, with an average of 92.42%. This underscores the fact that Cd is the primary contaminant present in soils surrounding the smelter.

**Figure 3:**
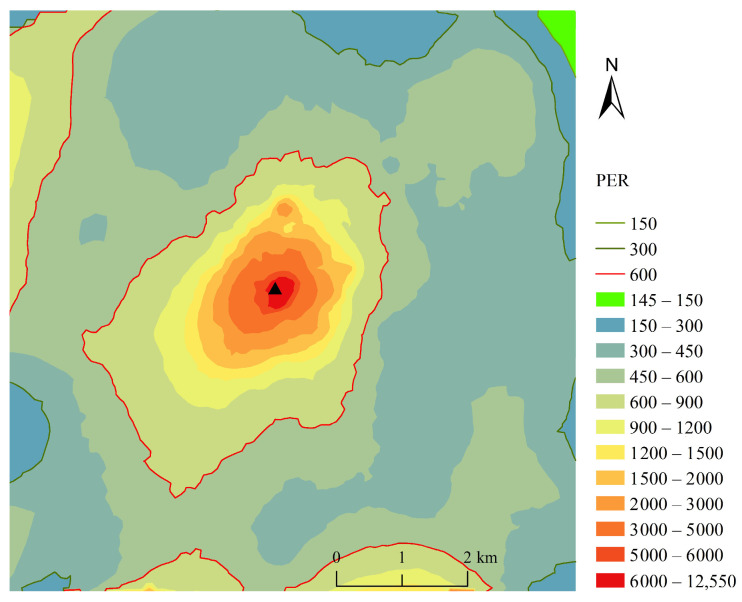
Distribution of grades on PER of heavy metal(oid)s in soil. (The black triangle represents the location of the smelter.)

According to the spatial distribution of PER around the smelter (Figure 3), the northeast corner of the study area is a low potential ecological risk area, with the PER less than 150, accounting for 0.44% of the total area. This region is situated at a considerable distance from the smelter and has no other industrial facilities in its vicinity. In contrast, moderate, high, and significantly high potential ecological risk areas accounted for 5.71%, 68.78%, and 25.07% of the study area, respectively, as illustrated in Table 6. The spatial distribution of PER reveals that the areas with significantly high potential ecological risk are primarily concentrated in an oval-shaped region within a 1.5 km radius of the smelter, which closely resembles the spatial distribution of Cd in the study area. Furthermore, the polluted soils with significantly high and high PER covered approximately 93.84% of all the investigated regions, as shown in Figure 3.

### 3.4. Multivariate Analysis

#### 3.4.1. PCA

**Figure 4:**
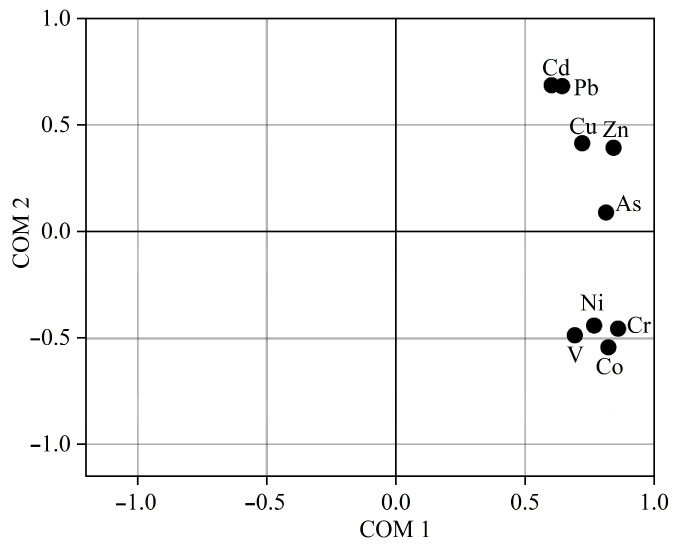
PCA loading 2-D plot (PC1 vs. PC2) for nine heavy metal(oid)s.

The findings of the PCA are presented in Figure 4. The primary component, which accounts for 60.08% of the overall variance, can be attributed to anthropogenic activities, as evidenced by the substantial factor loadings of Cd, Pb, Cu, and Zn. It is worth noting that Cd, Pb, and Zn are frequently co-present in Pb/Zn ores and are easily emitted during the smelting process [51]. The secondary component explains 26.26% of the total variance, with notable factor loadings of Co, Cr, Ni, and V. The prominent factor loadings of Cr and Ni are indicative of other industrial sources rather than the smelter. Given that lithogenic Cr and Ni are commonly found in primary minerals, the second component may signify the soil’s primary mineral factor.

Based on the PCA outcomes (Figure 4), in the coordinate system established by principal component 1 and principal component 2, the cluster of elements comprising Pb, Cd, Zn, Cu, and As is evidently distinguished from the cluster of Co, Cr, Ni, and V. It can be inferred that the former elements present in the topsoil are subject to anthropogenic influences, in addition to natural factors, while the latter elements are found to be in proximity to the natural background values in the corresponding region [52].

#### 3.4.2. Cluster Analysis (CA)

**Figure 5:**
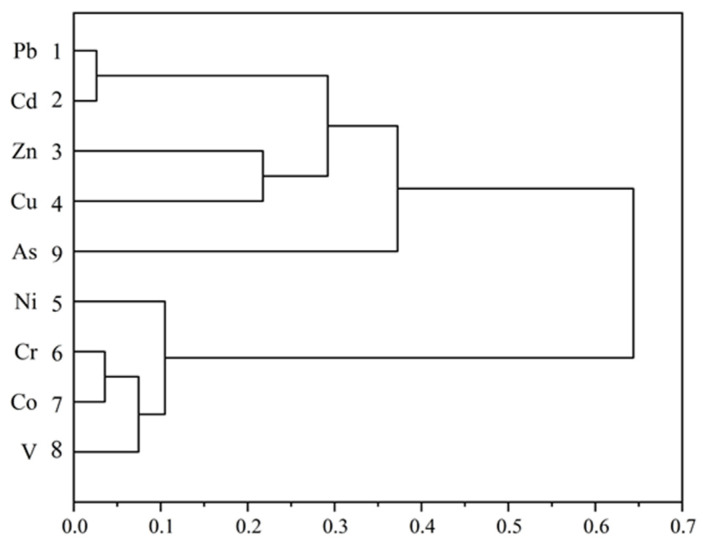
Hierarchical clustering of heavy metal(oid)s in soil near the smelter.

A CA was conducted to validate the outcomes of the PCA on heavy metal(oid)s (Figure 5). Generally speaking, the results of the CA demonstrated a high degree of agreement with those of the PCA analysis [53]. Two distinctive clusters were identified in the dendrograms for these metals in soils. Cluster I consisted of Pb-Cd-Zn-Cu-As, which comprised three sub-clusters: Pb-Cd, Zn-Cu, and As. Cu, Pb, and Zn are frequently associated elements and are normally detected in industrial emissions, suggesting that these elements have multiple anthropogenic/lithogenic sources [54]. All of these metals exhibited mean concentrations that were significantly higher than the background levels near the smelter. Cluster II included Cr-Co-V-Ni and only one sub-cluster. Significantly dissimilar Euclidean distances were observed between the two integrated clusters, possibly indicating different sources. The findings presented in Section 3.2 suggest that Pb, Cd, Zn, Cu, and As in the soil within the study area were primarily released by smelters, while Cr, Co, V, and Ni were mainly influenced by traffic, and fertilizer and other types of industrial plants. The results in this study are similar to the two PCA clusters in the southern part of the Nile Delta [23].

## 4. Conclusions

The soil in the study area was severely contaminated with heavy metal(oid)s, with an over-standard rate of 94.89% for Cd, 8.03% for Pb, and 10.22% for As. According to the distribution of different heavy metal(oid)s in soils, the concentration of Cd and Pb in soil decreases gradually with the increase of distance from the smelter to the surrounding area, showing an oval distribution, which accords with the typical air pollution diffusion model. Moreover, the distribution of Zn, Cu, and As was similar to Cd and Pb. However, Ni, V, Cr, and Co concentrations were mainly effected by factories and the soil parent materials. The potential ecological risk of Cd was higher than that of the other elements, mainly due to the extremely high risk while the risk grades of the other eight elements had a generally low risk. The polluted soils with significantly high and high potential ecological risk covered 93.84% of all the studied regions. Through PCA and CA analysis, it was found that Pb, Cd, Zn, Cu, and As were mainly attributable to human factors, via smelter emissions, with a contribution rate of 60.08%. Conversely, Co, Cr, Ni, and V are mainly caused by nature, with a contribution rate of 26.26%.

## Figures and Tables

**Table 1:** Risk grade indexes and grades of PER of heavy metal pollution.

E_r_	Risk Grade	PER	Risk Grade
<40	Low potential ecological risk	150	Low potential ecological risk
40–80	Moderate potential ecological risk	150–300	Moderate potential ecological risk
80–160	Considerable potential ecological risk	300–600	High potential ecological risk
160–320	High potential ecological risk	>600	Significantly high potential ecological risk
320	Significantly high potential ecological risk		-

**Table 2:** Descriptive statistic of selected soil chemical properties (*n* = 137).

	pH	TN (%)	OM (%)	CEC (cmol/kg)	Fe (g/kg)
Mean	8.56	0.21	2.56	22.91	26.03
SD	0.16	0.14	0.80	4.69	6.95
Median	8.56	0.18	2.55	22.82	26.33
Kurtosis	0.26	9.44	6.50	0.02	0.10
Skewness	−0.07	2.54	1.69	−0.16	0.28
Min	8.13	0.02	0.76	9.35	11.03
Max	9.11	0.96	6.32	32.39	49.78
CV(%)	1.89	68.41	31.11	20.48	0.03

SD: standard deviation; CV: coefficient of variation; OM: organic matter content; CEC: cation exchange capacity; TN: total nitrogen.

**Table 3:** Descriptive statistic of heavy metal(oid)s contents in the soils (*n* = 137).

	As	Cd	Co	Cr	Cu	Ni	Pb	V	Zn
Mean (mg/kg)	17.34	2.6	15.97	101.07	18.28	9.31	67.26	45.95	72.18
SD (mg/kg)	6.59	4.29	3.91	24.94	12.76	4.19	112.84	16.06	28.94
Median (mg/kg)	16.96	1.21	16.19	103.48	15.04	9.34	30.95	47.57	64.26
Min (mg/kg)	5.91	0.32	7.06	42.02	4.92	0.25	9.33	8.57	23.29
Max (mg/kg)	49.07	30.36	27.32	175.95	87.93	20.9	746.55	92.6	173.86
CV (%)	37.99	165.18	24.49	24.67	69.81	45.06	167.77	34.96	40.1
Background value ^a^ (mg/kg)	11.4	0.074	10	63.8	19.7	26.7	19.6	58.4	60.1
Exceeded background (%)	83.94	100.00	93.43	93.43	26.28	0	85.4	23.36	59.85
Screening values ^b^ (mg/kg)	25	0.6	-	250	100	190	170	-	300
Exceeded screening (%)	10.22	94.89	-	0	0	0	8.03	-	0

SD: standard deviation; CV: coefficient of variation. ^a^ Background values of soil elements in Henan Province [49]. ^b^ The risk screening values for agricultural land are cited from the national standard of China (GB 15618-2018).

**Table 4:** Descriptive statistic of E_r_ for individual metal(oid)s and PER for all metal(oid)s (*n* = 137).

E_r_	Range	Mean	SD
As	5.19–43.05	15.21	5.78
Cd	128.71–12,306.20	1054.16	1741.21
Co	3.53–13.66	7.98	1.95
Cr	1.32–5.52	3.17	0.78
Cu	1.25–22.32	4.64	3.24
Ni	0.06–4.7	2.09	0.94
Pb	2.38–190.45	17.16	28.79
V	0.29–3.17	1.57	0.55
Zn	0.39–2.89	1.2	0.48
PER	146.86–12,549.81	1107.18	1776.33

**Table 5:** Grade of potential ecological risk (E_r_) for each heavy metal(oid)s (%).

Heavy Metal	Low Risk	Moderate Risk	Considerable Risk	High Risk	Very High Risk
As	98.54	1.46	0	0	0
Cd	0	0	0.73	15.33	83.94
Co	100	0	0	0	0
Cr	100	0	0	0	0
Cu	100	0	0	0	0
Ni	100	0	0	0	0
Pb	91.97	2.19	5.11	0.73	0
V	100	0	0	0	0
Zn	100	0	0	0	0
PER	0.73	8.03	51.09	40.15	-

**Table 6:** The area cover ratio of different PER risk grades.

PER	Risk Grade	Cover Ratio %
150	Low potential ecological risk	0.44
150–300	Moderate potential ecological risk	5.71
300–600	High potential ecological risk	68.78
>600	Significantly high potential ecological risk	25.07

## Data Availability

Not applicable.
